# Hormonal and nutritional biomarkers in immune checkpoint inhibition for gastrointestinal cancers: a narrative review

**DOI:** 10.3389/fimmu.2026.1968875

**Published:** 2026-10-22

**Authors:** Hao Luo, Han Wu, HaiYu Zhang

**Affiliations:** 1 Department of Clinical Laboratory, Sichuan Clinical Research Center for Cancer, Sichuan Cancer Hospital & Institute, Sichuan Cancer Center, University of Electronic Science and Technology of China, Sichuan, China; 2 Department of Transfusion, Sichuan Clinical Research Center for Cancer, Sichuan Cancer Hospital & Institute, Sichuan Cancer Center, University of Electronic Science and Technology of China, Sichuan, China

**Keywords:** gastrointestinal tumors, hormone biomarkers, immune checkpoint inhibitors, nutritional biomarkers, precision immunotherapy, tumor immune microenvironment

## Abstract

Immune checkpoint inhibitors (ICIs) have improved treatment outcomes in selected gastrointestinal cancers, although the efficacy and frequency of immune-related adverse events (irAEs) vary substantially. Established tumor biomarkers, particularly microsatellite instability-high/mismatch repair deficiency (MSI-H/dMMR), guide treatment selection, whereas the additional value of circulating hormonal and nutritional markers remains uncertain. In this narrative review, we have examined the prognostic relevance of glucocorticoids, thyroid and sex hormones, albumin, vitamin D, and trace elements in gastrointestinal cancers based on clinical evidence. Albumin-based indices have shown prognostic value in gastric cancer, whereas cortisol and composite host-state scores require independent prospective evaluation. Furthermore, treatment-emergent thyroiditis is a toxicity-associated event with possible pharmacodynamic implications and should not be conflated with pretreatment thyroid function. Vitamin D has context-dependent immunoregulatory effects, and observational or preclinical findings do not establish that supplementation improves the efficacy of ICIs against gastrointestinal tumors. The evidence for zinc is exploratory at present, and clinical evidence for selenium remains insufficient. Tumor metabolic reprogramming provides a mechanistic connection between nutrient availability and immune function, but circulating markers cannot be considered definitive indicators of intra-tumoral metabolism. Microbiota-derived metabolites provide a potential connection between nutrient availability, endocrine signaling, and immune function, although their contribution to clinical ICI benefit remains incompletely defined. These biomarkers may guide supportive assessment and research stratification, but do not justify withholding otherwise indicated ICI therapy, diagnosing resistance, or altering oncological surveillance. Clinical translation requires standardized assays, appropriate comparators, treatment–biomarker interaction testing, and validated models with demonstrated clinical utility.

The roles of hormonal and nutritional biomarkers in gastrointestinal cancer immunotherapy with ICIs. Hormones (GC, estrogen, androgen, T3/T4) modulate immune cells via PD-L1 regulation. Nutrients (albumin, vitamin D, trace elements) shape anti-tumor immunity and immunometabolism. Their interactions may contribute to heterogeneity in host status and treatment outcomes. Current clinical use centers on supportive assessment and toxicity monitoring; prognostic modeling and interventions intended to modify ICI efficacy require prospective validation.

## 1 Introduction

Gastrointestinal malignancies, including gastric, colorectal, and esophageal cancers, encompass diseases with distinct molecular profiles and treatment pathways. Immune checkpoint inhibitors (ICIs) targeting programmed death-1 (PD-1), programmed death-ligand 1 (PD-L1), or cytotoxic T-lymphocyte-associated protein 4 (CTLA-4) have expanded therapeutic options in selected settings. Randomized trials support pembrolizumab in microsatellite instability-high/mismatch repair deficiency (MSI-H/dMMR) metastatic colorectal cancer (CRC), nivolumab plus chemotherapy in advanced gastric/gastroesophageal junction and esophageal adenocarcinoma, and nivolumab-based combinations in advanced esophageal squamous-cell carcinoma (1–3). Nevertheless, these indications should not be generalized across all gastrointestinal tumors or treatment lines.

In June 2020, the US Food and Drug Administration approved pembrolizumab for first-line treatment of unresectable or metastatic MSI-H/dMMR CRC, with tumor MSI/MMR status established using appropriate testing (4). In the KEYNOTE-177 trial conducted on 307 MSI-H/dMMR CRC patients, the median progression-free survival (PFS) was 16.5 months in the pembrolizumab arm versus 8.2 months in the chemotherapy arm (hazard ratio [HR], 0.60; 95% confidence interval [CI], 0.45–0.80) (1). In an earlier phase II study, only dMMR CRC patients responded to pembrolizumab, and no response was observed in a small mismatch repair-proficient (pMMR) CRC cohort (5). Consequently, the associations derived from MSI-H/dMMR disease cannot be transferred to microsatellite-stable/pMMR (MSS/pMMR) disease without specific evidence. More recent studies reinforce the importance of disease setting and molecular selection. In the randomized CheckMate 8HW trial, 303 previously untreated patients were assigned to nivolumab plus ipilimumab or chemotherapy; the primary efficacy population comprised of 255 patients with centrally confirmed MSI-H/dMMR tumors (6). In contrast, NICHE-2 was a single-arm phase II study of neoadjuvant nivolumab plus ipilimumab in non-metastatic dMMR colon cancer: 115 patients were enrolled, and 75 of 111 efficacy-evaluable patients had a pathological complete response (7). Neither study established a circulating hormone or nutritional threshold for treatment selection, and their findings should not be extrapolated to unselected MSS/pMMR CRC.

Hormonal homeostasis, inflammation, nutritional intake, and body composition may influence treatment tolerance and prognosis. Nevertheless, a circulating analyte reflects disease burden, organ dysfunction, medication exposure, or systemic inflammation rather than being a causal determinant of anti-tumor immunity. In this review, we have evaluated whether host biomarkers offer information beyond established tumor features and clinical status, while separating biological mechanisms, clinical associations, and intervention effects. Gastrointestinal studies and relevant evidence from other cancers have been distinguished throughout the review.

### 1.1 Review scope and literature identification

This is a narrative review with a targeted evidence update completed on 20 September 2026. MEDLINE/PubMed records were retrieved through Europe PMC and checked against PubMed Central full texts or original publisher reports when available; FDA publications were consulted for regulatory indications. Search keywords included combinations of gastric, colorectal, esophageal, checkpoint, nivolumab, pembrolizumab, cortisol, thyroid, albumin, prognostic nutritional index, vitamin D, zinc, selenium, metabolic reprogramming, sarcopenia, microbiome, antibody clearance, and prediction-model validation. Additional searches prioritized publications from 2024 through 20 September 2026, while retaining directly relevant earlier studies. For example, the Europe PMC query TITLE_ABS:(gastric nivolumab albumin) AND SRC: MED was used to identify gastric cancer studies; targeted title and DOI searches were performed to verify specific publications. Foundational mechanistic studies were retained without a lower publication-date limit, and relevant references in the retrieved articles were examined manually.

Primary clinical studies published in English were prioritized for outcome claims. Mechanistic experiments, professional guidance, and selected reviews were used for biological context or clinical interpretation. Selection was based on relevance to the stated biomarker, cancer population, treatment, and endpoint, rather than statistical significance. Duplicate records and citations unrelated to the accompanying assertion were removed. We did not perform an exhaustive systematic search, did not use independent duplicate screening or a registered protocol, and did not generate pooled estimates. Selected clinical evidence and model validation, including missing information and reporting inconsistencies, have been summarized in **Tables 1** and **2**.

**Table 1:** Selected clinical evidence for host biomarkers and related nutritional or microbiome interventions.

Study and population	Design, treatment and sampling	Biomarker/molecular context	Outcome estimate (95% CI)	Comparator and principal limitations
Zhao et al., 2026 (24) Gastric cancer; n=134	Retrospective, single center. Mixed anti-PD-1 regimens, mostly chemotherapy combinations. Fasting baseline cortisol, 06:00–08:00.	Cortisol cutoff 193.95 nmol/L (data-derived). **Table 1**: MSS/MSI-L 112; MSI-H 6; unknown 16.	High vs low: adjusted OS HR 2.027 (1.053–3.901); PFS HR 1.639 (0.999–2.686). Estimates from source Table 4.	No non-ICI comparator or interaction test. Heterogeneous regimens; incomplete molecular data. Abstract/text and Table 4 differ in OS CI; other reporting inconsistencies.
Sato et al., 2021 (16) Gastric cancer; n=278	Retrospective, multicenter. Later-line nivolumab monotherapy; median 4 cycles. Pretreatment albumin.	Albumin >3.5 vs ≤3.5 g/dL. Differentiated/undifferentiated histology included; MSI and PD-L1 not evaluated.	Adjusted OS HR 0.688 (0.478–0.991) for higher albumin. Multivariable analysis included inflammatory, clinical, histological and irAE variables.	No non-ICI comparator or interaction test. Data-derived cutoff; residual confounding. Treatment-emergent irAE adjustment raises timing concerns.
Lee et al., 2022 (40) Gastric cancer with peritoneal metastases; n=35	Retrospective, single center. Nivolumab 3 mg/kg every 2 weeks, predominantly second/third line. Baseline PNI.	PNI ≥40 vs <40. MSI/MMR and PD-L1 not reported for stratified estimates.	Higher PNI: adjusted PFS HR 0.366 (0.155–0.861); OS HR 0.349 (0.142–0.860). Adjusted for age, histological differentiation and ECOG PS.	No comparator or interaction test. Small selected cohort; median follow-up 2 months; no objective responses. PNI includes lymphocytes as well as albumin.
Takegawa et al., 2023 (17) Esophageal cancer; 37 nivolumab, 35 taxane	Retrospective historical comparison. Nivolumab 240 mg every 2 weeks after fluoropyrimidine/platinum; pretreatment laboratory tests.	PNI <45 vs ≥45; albumin <3.5 vs ≥3.5 g/dL. SCC: 34/37 nivolumab and 31/35 taxane; MSI/PD-L1 not reported.	Nivolumab cohort: unadjusted OS HR 2.725 (1.249–5.947) for low PNI; 4.406 (1.502–12.928) for low albumin. Adjusted estimates not reported.	Historical taxane comparator; no formal treatment–marker interaction. Calendar-period differences and unequal prior treatment; small sample and unadjusted analyses.
Wang et al., 2025 (54) Mixed tumors; n=98 (47 digestive, 51 lung)	Single-center observational cohort; 68 training and 30 temporal validation. PD-(L)1 inhibitors plus chemotherapy; baseline T0 and pre-second-dose T1.	Association at T1; baseline concentrations did not separate benefit. Threshold 14.2; units conflict (μg/L vs μg/mL). GI molecular subtypes not reported.	Training sensitivity 100%, specificity 41.86%; validation true-positive/negative rates 73%/37%. GI-specific adjusted HRs with CIs not reported.	No non-ICI comparator or interaction test. Small mixed population; treatment-line imbalance, postbaseline selection, unclear units. Same-center temporal validation only.
Street et al., 2021 (21) Pan-cancer; n=6,596	Retrospective. Mostly PD-(L)1 monotherapy; CTLA-4 or combination also included. Baseline thyroid disease excluded. Thyroiditis developed in 1,155 patients.	Treatment-emergent thyroiditis; median onset 3.1 months. No pretreatment selection threshold or GI-specific molecular estimate.	Adjusted OS HR: time-independent 0.47 (0.44–0.53); time-varying 0.80 (0.71–0.89). Six-month landmark HR 0.76 (0.65–0.88).	No non-ICI comparator or interaction test. Marked attenuation after addressing immortal time bias; residual confounding and heterogeneous cancers. Indirect GI evidence.
Galus et al., 2023 (48) Advanced melanoma; n=200 (199 classified)	Observational: historical unsupplemented and prospectively supplemented cohorts. First-line nivolumab/pembrolizumab. 25(OH)D at baseline and 9–12 weeks.	Reduced 25(OH)D ≤30 ng/mL vs normal at baseline or achieved after supplementation. No GI cohort.	ORR 56.0% vs 36.2%; adjusted PFS HR 0.65 (0.49–0.94) for normal status. OS difference not significant.	No randomized supplementation comparison or ICI-versus-other-treatment interaction. Calendar and postbaseline classification bias; does not establish GI efficacy.
Grover et al., 2020 (50) Melanoma; discovery n=213, validation n=169	Retrospective cohorts. Anti-PD-1, anti-CTLA-4 or combination therapy. Supplement use recorded before ICI initiation.	Vitamin D exposure history, not a serum cutoff. Colitis: 37/213 and 49/169. No GI-specific population.	Adjusted OR for ICI colitis: 0.35 (0.1–0.9) in discovery; 0.46 (0.2–0.9) in validation.	Supplement users vs nonusers; no randomization or treatment–marker interaction. Toxicity association, not antitumor efficacy or proven preventive effect.
Wang X et al., 2023 (41) Advanced gastric adenocarcinoma; n=195	Retrospective, single center. Anti-PD-1 plus chemotherapy (166), monotherapy (24), or apatinib (5). Baseline blood within 3 days before treatment; ≥3 cycles required.	HER2-negative; pMMR 178, dMMR 17. PNI median split 45.5; distinct response-derived ROC cutoff 47.18.	Baseline PNI: adjusted PFS HR 0.949 (0.924–0.975) per unit increase. Model included PD-L1 and MMR status.	No non-ICI comparator or interaction test. Mixed treatment lines/regimens. Cycle-based eligibility selects patients remaining on treatment. Cutoffs not externally validated.
Wang B et al., 2025 (42) Metastatic/recurrent or unresectable ESCC; n=677	Retrospective, single center. Anti-PD-1 alone (92), plus chemotherapy (324), radiotherapy (97), or both (164). Pretreatment GNRI; >2 cycles required.	GNRI cutoff 91.5 (time-dependent ROC-derived). PD-L1 assessed in a selected 45-patient subset; no molecularly stratified primary analysis.	High vs low GNRI: adjusted OS HR 0.648 (0.532–0.789) in the full cohort.	No non-ICI comparator or treatment interaction. GNRI correlated with performance status and treatment modality; residual confounding and survival-based eligibility.
Deng et al., 2024 (58) Gastric cancer; n=115	Retrospective. Mixed PD-(L)1-containing regimens; individual regimen details insufficiently specified. Pretreatment L3 CT muscle measurements.	SMI cutoffs 27.36 cm²/m² (men), 31.10 (women), data-derived. Myosteatosis: density <41 HU if BMI <25, <33 HU otherwise. MSI/MMR not reported.	Adjusted OS HR: sarcopenia 3.569 (1.808–7.045); myosteatosis 3.172 (1.471–6.839).	No non-ICI comparator or interaction. Small heterogeneous cohort, incomplete tumor-marker data, nonstandard muscle cutoffs; no intervention inference.
Tao et al., 2024 (59) Digestive cancers; n=98 (CRC 51, gastric 36, esophageal 4, other 7)	Retrospective. Mixed immunotherapy regimens. Cachexia classified using weight or psoas-area changes from treatment start to ≥2 cycles.	On-treatment cachexia assessment; not a wholly baseline exposure. CRC MSI/MMR-specific results not reported.	Cachexia–OS association: log-rank P = 0.027; cachexia–PFS comparison P = 0.321. Adjusted cachexia-specific HR/CI not reported in the multivariable table.	No non-ICI comparator or interaction. Small, mixed cancers; reassessment-dependent inclusion creates time-related selection. Albumin estimates must not be attributed to cachexia.
Vemulapalli et al., 2026 (51) Mixed cancers with immune-mediated colitis; n=179	Retrospective, single center. Mixed ICI regimens, most commonly pembrolizumab. Vitamin D measured within 6 months before or after ICI initiation.	Serum vitamin D ≤40 vs >40 ng/mL; 121 vs 58 patients. Threshold is study-specific, not a standard deficiency definition.	Grade ≥2 colitis: 48.0% vs 28.6% (P = 0.034). CI and adjusted effect estimate for this severity comparison not reported.	Only colitis cases included: cannot assess incidence. Broad sampling window, reverse causation, supplementation confounding; no GI-specific antitumor endpoint.
Ng et al., 2026; SOLARIS (49) Previously untreated metastatic CRC; n=455	Double-blind phase III RCT. mFOLFOX6 or FOLFIRI plus bevacizumab; NO ICI. High-dose vs standard-dose vitamin D3 from treatment initiation.	High dose: 8,000 IU/day for 14 days, then 4,000; control: 400 IU/day. Not selection by an ICI-specific serum vitamin D threshold.	Median PFS 11.8 months (10.3–13.3) vs 10.3 (9.4–12.2); one-sided P = 0.25. Primary endpoint not improved.	Randomized supplementation comparator, but no ICI arm. Cannot estimate a supplementation–ICI interaction or establish effects in an MSI/MMR-defined ICI population.
Zhang et al., 2026 (91) ICI-refractory GI cancer; n=10 (gastric 8, CRC 2)	Single-arm phase I. Initial oral healthy-donor FMT, then maintenance FMT plus nivolumab 3 mg/kg every 2 weeks for 6 cycles; serial stool/blood sampling.	Report focuses on MSS gastric disease. Exploratory microbial/immune signatures; no validated pretreatment threshold.	ORR 2/10 (20%); both responses in gastric cancer. Response CI not reported; no comparative adjusted efficacy estimate.	No control or interaction test; very small mixed cohort. Feasibility and exploratory response signals, not proof of resistance reversal or survival benefit.
Zhao et al., 2023/2024; RENMIN-215 (79, 80) Refractory MSS metastatic CRC; n=20	Single-arm phase II after ≥2 prior lines. FMT plus tislelizumab and fruquintinib. Follow-up included an exploratory real-world comparison.	All MSS/pMMR. Serial microbial profiling; no validated metabolite or hormone threshold.	ORR 20.0% (5.7–43.7); median PFS 9.6 months (4.1–15.1). Follow-up: matched OS HR 0.26 (0.07–0.95); PFS difference not significant.	94 controls screened; only 10 trial patients matched to 16 controls. Residual selection/confounding; antiangiogenic treatment prevents isolation of FMT efficacy.

Selected clinical evidence; these studies do not establish differential ICI benefit. CI, confidence interval; ECOG PS, Eastern Cooperative Oncology Group performance status; GI, gastrointestinal; HR, hazard ratio; ICI, immune checkpoint inhibitor; irAE, immune-related adverse event; MSI-H, microsatellite instability-high; MSI-L, microsatellite instability-low; MSS, microsatellite-stable; OR, odds ratio; ORR, objective response rate; OS, overall survival; PFS, progression-free survival; PNI, prognostic nutritional index; SCC, squamous-cell carcinoma. PNI = 10 × albumin (g/dL) + 0.005 × lymphocyte count (/μL). “Not reported” indicates that the stated information was not available in the reviewed report; no estimate has been imputed. CT, computed tomography; dMMR, deficient mismatch repair; ESCC, esophageal squamous-cell carcinoma; FMT, fecal microbiota transplantation; GNRI, geriatric nutritional risk index; HU, Hounsfield units; pMMR, proficient mismatch repair; RCT, randomized controlled trial; SMI, skeletal muscle index. The SOLARIS row describes a non-ICI supplementation trial and is included to distinguish randomized nutritional-intervention evidence from evidence of ICI sensitization.

**Table 2:** Reported performance and validation of selected host-biomarker models.

Model/source and intended outcome	Inputs, population and treatment	Discrimination	Calibration and validation	Interpretation and remaining limitations
Sato pretreatment score (16) OS stratification	n=278 gastric cancer; later-line nivolumab. Five baseline factors: CRP, albumin, ECOG PS, lymphocyte count and histology. irAEs excluded from pretreatment score.	Survival separation reported across score groups. Model C-index and numerical AUC with CI not reported.	Formal calibration not reported. Multicenter development cohort; no independent external validation.	Data-derived thresholds and retrospective selection. Separate six-factor analysis included irAE occurrence and is unsuitable as a wholly baseline model. No treatment comparator or interaction test.
Wang zinc/CRP nomograms (54) PFS at 6/10 months; OS at 12/18 months	n=98 mixed lung/digestive tumors; ICI plus chemotherapy. T1 zinc and CRP; training 68, temporal validation 30.	Main report does not provide a numerical nomogram C-index/AUC with CI. Zinc classification results are summarized in **Table 1**.	Calibration plots presented; slope/intercept not reported. Same-center temporal cohort; no independent-center validation.	Postbaseline sampling; inconsistent zinc units; no GI-specific validation or non-ICI comparator. Prognostic tools remain exploratory.
Li ENI-Score, 2026 (85) Major pathological response (≤10% viable tumor)	Retrospective gastric/GEJ adenocarcinoma, n=738: training 335, temporal 114, external 289. Morning cortisol, PNI, prealbumin, NLR, CRP/albumin. Neoadjuvant PD-1 + SOX/XELOX/FLOT/DOS; median 4 cycles. MSI-H/dMMR 54/738.	AUC: training 0.873, internal 0.816, external 0.830 (95% CI, 0.78–0.87). Adjusted MPR OR per 10 points 1.72 (1.59–1.86).	External Brier score 0.170; slope 0.90. Overall intercept 0.16, slope 0.94; Hosmer–Lemeshow P = 0.026. External centers n=3; slopes 0.68–1.15.	Algorithm selection considered external AUC, limiting validation independence. Surgical completers only; highly correlated inputs and mixed regimens. No ICI-free comparator or treatment–score interaction; no prospective impact study.

AUC, area under the receiver operating characteristic curve; CRP, C-reactive protein; ENI, Endocrine–Nutritional Immunotherapy response; GEJ, gastroesophageal junction; MPR, major pathological response; NLR, neutrophil-to-lymphocyte ratio. SOX, S-1/oxaliplatin; XELOX, capecitabine/oxaliplatin; FLOT, fluorouracil/leucovorin/oxaliplatin/docetaxel; DOS, docetaxel/oxaliplatin/S-1. Model performance describes outcome prediction in the studied population and does not establish prediction of incremental ICI benefit or clinical utility.

### 1.2 Biomarker definitions and disease context

A prognostic biomarker is associated with the likelihood of an outcome in a defined clinical setting, while a predictive biomarker identifies differences in the effect of a treatment relative to an alternative. A pharmacodynamic/response biomarker reflects a biological change after exposure, whereas a safety biomarker is a measure of toxicity (8). A favorable survival association within an ICI-treated cohort is therefore insufficient to establish ICI-specific predictive value. That inference generally requires an appropriate comparator and a treatment–biomarker interaction analysis. Thyroiditis is an adverse event rather than a pre-treatment selection test; its laboratory indices can be used for toxicity monitoring and as pharmacodynamic correlates.

Study interpretation must account for histology, stage, molecular subtype, treatment line, combination partners, dosing schedule, and sampling relative to each cycle. Studies on gastric and gastroesophageal junction adenocarcinoma patients should report HER2, PD-L1 combined positive score (CPS), and MSI/MMR status, as well as Epstein–Barr virus status when available. Furthermore, esophageal squamous-cell carcinoma should be distinguished from adenocarcinoma. The CheckMate 649 trial evaluated nivolumab plus fluoropyrimidine–oxaliplatin chemotherapy on two- or three-week schedules in patients with non-HER2-positive adenocarcinoma, whereas CheckMate 648 examined distinct nivolumab combinations in a squamous-cell carcinoma cohort (2, 3). Host-marker associations obtained during later-line nivolumab monotherapy cannot be presumed equivalent to those identified during first-line or perioperative immunochemotherapy.

The regimen-specific evaluation of host biomarkers has been demonstrated in contemporary gastric cancer trials. In the 4.5-year follow-up of KEYNOTE-859, Rha et al. reported an OS HR of 0.78 (95% CI, 0.70–0.86) for pembrolizumab plus chemotherapy versus chemotherapy in 1579 patients with untreated, advanced HER2-negative gastric/GEJ adenocarcinoma (9). The final KEYNOTE-811 analysis involved 698 patients with HER2-positive disease receiving trastuzumab and chemotherapy; adding pembrolizumab improved OS (HR, 0.80; 95% CI, 0.67–0.94) (10). The RATIONALE-306 trial evaluated first-line tislelizumab plus a platinum-based doublet every three weeks in 649 patients with esophageal squamous-cell carcinoma, with an OS HR of 0.66 (95% CI, 0.54–0.80) versus chemotherapy alone (11). These different treatment backbones may affect intake, inflammation, endocrine function, and biomarker kinetics.

Perioperative studies represent another distinct setting. In the MATTERHORN trial, 948 patients with resectable gastric/GEJ adenocarcinoma were randomized to durvalumab or placebo with perioperative FLOT, followed by maintenance durvalumab or placebo; the event-free survival HR was 0.71 (95% CI, 0.58–0.86) (12). In contrast, the non-randomized phase II NEONIPIGA study enrolled 32 patients with localized MSI-H/dMMR gastric/GEJ adenocarcinoma to receive neoadjuvant nivolumab plus ipilimumab, surgery, and adjuvant nivolumab. Pathological complete response occurred in 17 of 29 patients who underwent resection (58.6%; 90% CI, 41.8–74.1) (13). Nutritional or endocrine associations in these settings require separate evaluation from that involving patients with heavily pretreated metastatic disease.

### 1.3 Confounding and the timing of biomarker assessment

Tumor stage and disease burden can influence both host biomarkers and ICI outcomes. Extensive disease may increase inflammatory catabolism and stress signaling, while liver involvement, gastrointestinal obstruction, or peritoneal disease can alter albumin synthesis, intake, or nutrient absorption (14, 15). An association between hypoalbuminemia and poor survival may therefore partly reflect disease severity. Analyses should distinguish resectable from metastatic disease and account for tumor burden, metastatic sites, and Eastern Cooperative Oncology Group performance status (ECOG PS), rather than attribute the entire association to impaired antitumor immunity.

Prior treatment also changes the biological meaning of a baseline measurement. Chemotherapy and radiotherapy may leave persistent marrow suppression or mucosal injury; previous gastrointestinal surgery can affect absorption and microbial ecology. Later-line cohorts additionally select patients who have survived earlier therapies and remain eligible for further treatment. Studies should record the number and type of previous treatment lines, the interval from the last treatment, prior surgery, and residual toxicity. Associations obtained during later-line nivolumab monotherapy (16, 17) require separate assessment from those observed during first-line immunochemotherapy (2, 3).

Medication ascertainment should extend beyond glucocorticoids. Antibiotics and proton pump inhibitors (PPIs) may alter microbial exposure, whereas thyroid medication, sex-steroid preparations, and vitamin or trace-element supplements directly affect the corresponding measurements. Metformin, nonsteroidal anti-inflammatory drugs, and opioids should also be recorded because their metabolic effects or prescribing indications may relate to host status. For each exposure, the relevant information includes indication, dose, start and stop dates, and timing relative to sampling and ICI initiation. These variables should be evaluated as potential confounders without assuming that every drug class has an established ICI-specific effect.

Gastric cancer data illustrate why exposure and indication cannot be reduced to a single binary variable. In 77 patients receiving later-line nivolumab, Shibano et al. associated peri-initiation PPI use with shorter PFS after multivariable adjustment (HR, 1.93; 95% CI, 1.03–3.64), but the adjusted OS association was not significant (18). In a separate 252-patient cohort, largely treated with immunochemotherapy, Zhang et al. observed longer OS with prophylactic antibiotic exposure than with no antibiotic exposure (HR, 0.57; 95% CI, 0.39–0.83); antibiotic use for infection represented a clinically different exposure (19). Treatment setting, infection severity, prior surgery, and residual confounding limit causal interpretation of both reports.

Covariates should be selected according to the biological question and their temporal position, preferably using a prespecified causal diagram, rather than univariable statistical significance alone (20). Inflammation, for example, may be a common cause of low albumin and poor outcome, or an intermediate on a hypothesized nutritional pathway. Adjustment for a mediator can obscure the total effect being investigated. Baseline association analyses should use information available at ICI initiation; medications or irAEs developing later require time-dependent or appropriately defined landmark analyses (21). Sensitivity analyses should examine treatment line, regimen, infection-related prescribing, and informative loss of follow-up samples.

## 2 Role of hormone biomarkers in ICI therapy for gastrointestinal tumors

Hormones participate in immune regulation and metabolic adaptation, but their circulating levels and tissue receptor activities are distinct biological indices. Endogenous hormone production, prescribed hormonal therapy, and ICI-induced endocrine injury also have different temporal relationships to treatment, and offer insights into clinical outcomes and the underlying mechanisms.

### 2.1 Glucocorticoids

Adrenal glucocorticoid (GC) secretion is regulated by the hypothalamic–pituitary–adrenal axis, and contributes to metabolic adaptation and the stress response. Endogenous cortisol, exogenous corticosteroids present at ICI initiation, and corticosteroids subsequently administered for immune-related adverse events (irAEs) must be analyzed separately. Baseline cortisol interpretation requires documentation of collection time, acute illness, binding-protein changes, liver function, and recent steroid exposure. Drug-related analyses additionally require prednisone-equivalent dose, route, duration, timing, and indication. **Figure 1** summarizes these distinct exposure categories.

**Figure 1:**
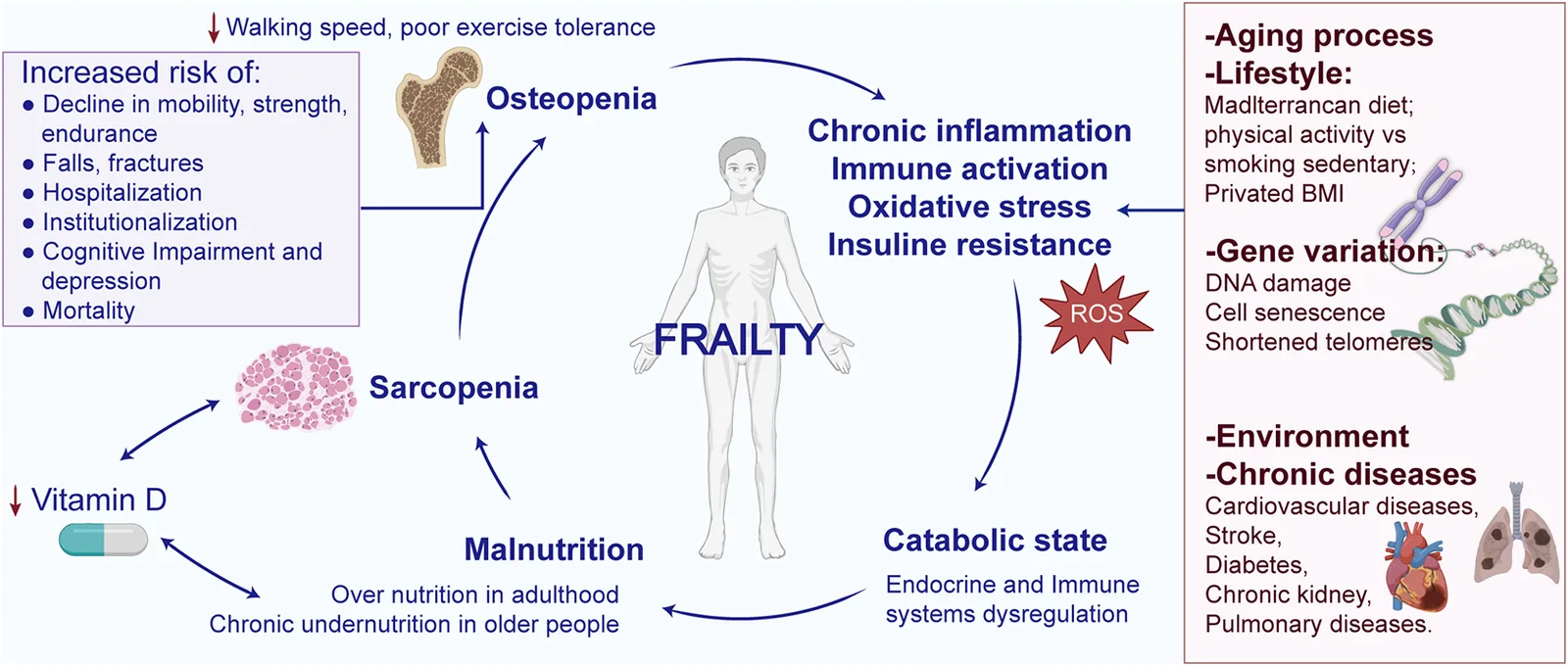
Immunomodulatory effects of glucocorticoids during inflammation and neoplasia. This original schematic illustrates the distinct effects of GCs during acute inflammation, chronic inflammation, and neoplasia. Although GCs may promote resolution of acute inflammation, sustained exposure can impair tissue repair and immune surveillance and may favor tumor persistence. In gastrointestinal cancers treated with ICIs, the clinical implications of GC exposure depend on dose, timing and indication, and should be distinguished from endogenous cortisol status and glucocorticoid use for irAEs.

Experimental studies support an immunoregulatory role for GC receptor signaling. Acharya et al. showed that glucocorticoids induced inhibitory receptors in CD8^+^ tumor-infiltrating T cells and impaired their differentiation, whereas receptor deletion led to improved tumor control in experimental models (22). Deng et al. linked tumor GC receptor activity to increased PD-L1 expression, reduced major histocompatibility complex class I (MHC I) expression, and resistance to checkpoint blockade in pancreatic ductal adenocarcinoma models (23). These findings provide mechanistic plausibility, but tissue-specific signaling cannot be inferred directly from a single measurement of serum cortisol level, and pancreatic experimental results do not establish a clinical cortisol threshold for gastric or colorectal cancer.

Direct evidence of glucocorticoid signaling in gastric cancer has recently emerged. Zhao et al. retrospectively studied 134 patients receiving heterogeneous anti-PD-1-based regimens; fasting cortisol was measured at 6 AM–8 AM before treatment, and their data-derived cutoff was 193.95 nmol/L. The adjusted overall survival (OS) HR for high cortisol group versus low cortisol group was 2.027 (95% CI, 1.053–3.901), and the corresponding adjusted PFS HR was 1.639 (95% CI, 0.999–2.686), indicating a borderline association. Nevertheless, incomplete MSI documentation, mixed treatments, reporting discrepancies, and absence of a non-ICI comparator limit interpretation (24). These results support a provisional prognostic association in gastric cancer, not a validated cortisol-based selection rule or an equivalent conclusion in CRC.

The evidence for exogenous corticosteroids largely comes from other malignancies. Arbour et al. evaluated 640 patients with non-small-cell lung cancer receiving single-agent PD-(L)1 blockade and observed worse outcomes in patients with baseline prednisone-equivalent doses of at least 10 mg/day (25). In a separate 650-patient lung cancer cohort, Ricciuti et al. found that adverse outcomes were more common among patients receiving corticosteroids for cancer-related palliation, although indication and disease severity confounded the results (26). These observations do not quantify the effect of physiological cortisol or prove that clinically necessary irAE treatment reduces the benefits of ICIs. Steroids for serious irAEs should be prescribed according to the severity of symptoms and established guidance, and the dose and timing should be recorded as time-varying exposures in outcome analyses (27).

Evidence concerning toxicity-directed corticosteroids should also be distinguished from baseline exposure. Verheijden et al. analyzed 834 patients who received immunosuppression for treatment-related adverse events across six trials of nivolumab plus ipilimumab, including CheckMate-142 and CheckMate-648 (28). A peak prednisolone dose of 2 mg/kg versus 0.5 mg/kg was associated with worse OS (age- and sex-adjusted HR, 1.66; 95% CI, 1.17–2.37), whereas cumulative dose was not associated with survival. Corticosteroid dose was not randomized, and the analysis does not isolate a gastrointestinal-specific causal effect. Toxicity severity and indication remain important confounders, and these findings do not justify delaying necessary irAE treatment. Treatment-emergent adrenal insufficiency provides a further distinction from baseline cortisol status. Researchers identified 20 cases among 1,014 ICI-treated patients in a mixed-cancer cohort, all of whom received corticosteroid replacement (29). The predominantly lung-cancer population and retrospective case selection limit gastrointestinal efficacy inferences, but support evaluation of new cortisol deficiency as endocrine toxicity.

### 2.2 Thyroid hormones

Thyroid hormones regulate metabolism, growth, and development, and can influence immune-cell activity. Thyroxine (T4) and triiodothyronine (T3) are thyroid products, whereas thyroid-stimulating hormone (TSH) is a pituitary regulator. A low TSH may accompany thyrotoxicosis, pituitary dysfunction, medication effects, or non-thyroidal illness, and does not by itself demonstrate impaired immunity. Interpretation should be based on paired TSH and free T4 measurements, symptoms, baseline values, and relevant medication history (30). **Figure 2** separates baseline abnormalities from treatment-emergent thyroid injury.

**Figure 2:**
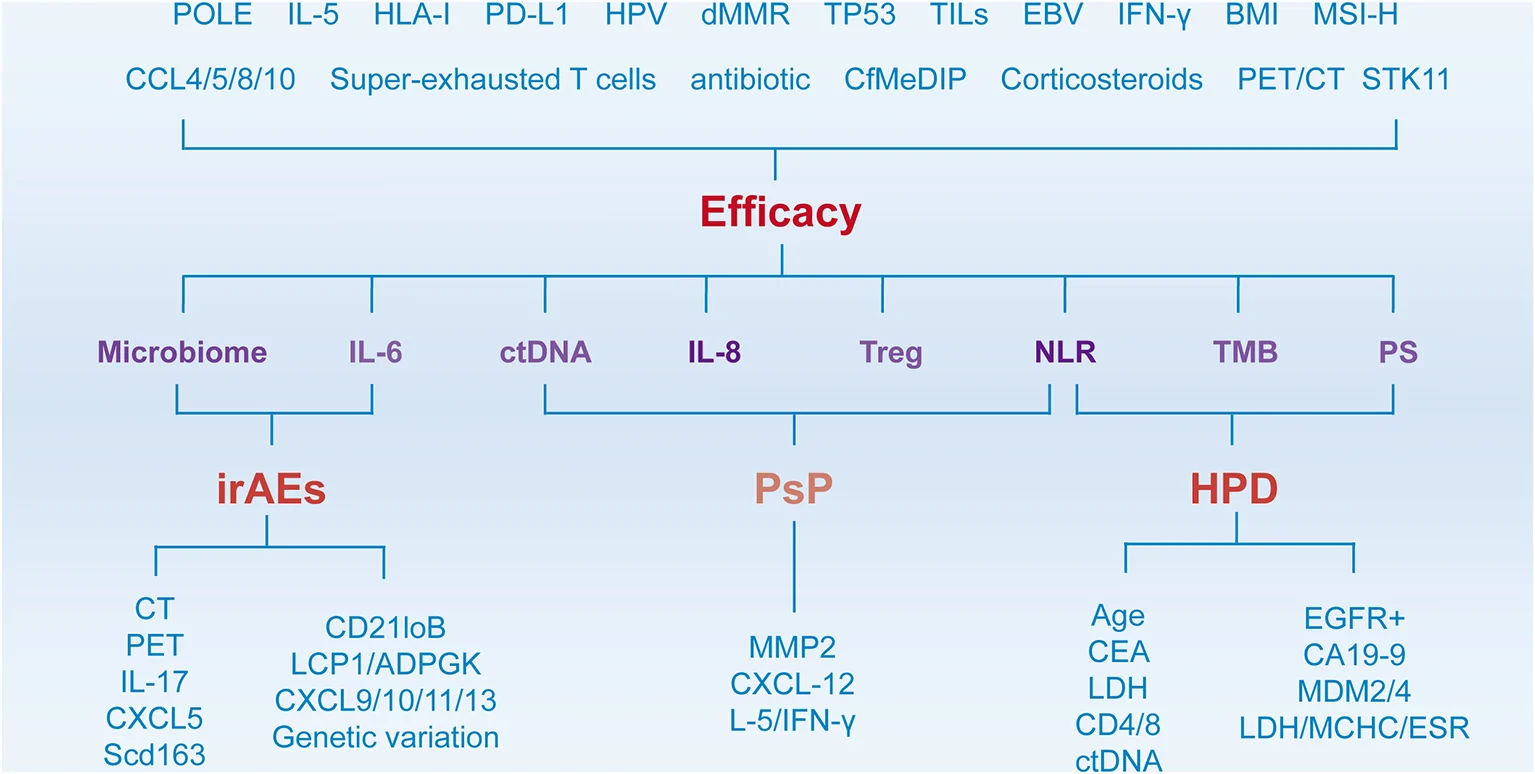
Proposed mechanisms of ICI-associated thyroid dysfunction in patients with gastrointestinal malignancies. ICI-associated cytotoxic T-cell activation may cause thyroid follicular-cell injury, followed by glandular atrophy or fibrosis and the development of hypothyroidism. Compensatory elevation of TSH may promote thyroid follicular hyperplasia, whereas B-cell activation and thyroid-stimulating hormone receptor antibodies (TRAb) may contribute to hyperthyroidism in a subset of patients.

Pre-treatment thyroid dysfunction describes a pre-existing clinical state that may influence symptoms and functional status. In contrast, ICI-induced destructive thyroiditis typically develops after exposure and may pass through a transient thyrotoxic phase before hypothyroidism. Central hypothyroidism associated with hypophysitis requires separate assessment, particularly for concomitant adrenal insufficiency (30). Neither a low baseline free T3 concentration during systemic illness, nor isolated TSH abnormality has been established here as a gastrointestinal ICI treatment-selection biomarker. The independent impact of normal baseline thyroid function or higher free T3 on the ICI response in gastric or colorectal cancer needs to be validated using directly applicable clinical data.

The consensus definitions published in 2025 further separate the thyrotoxic and hypothyroid phases of immune-related thyroiditis from Graves’ disease, hypophysitis, and adrenalitis (31). Applying these diagnosis-specific definitions is important when extracting endocrine events from records: a treatment-emergent low free T4 concentration with an inappropriately low or normal TSH cannot be grouped automatically with primary thyroid failure or a pretreatment low-T3 state.

Treatment-emergent thyroiditis has been associated with longer survival, but patients must survive long enough for the event to develop and be detected. Street et al. analyzed 6596 ICI-treated cancer patients without baseline thyroid disease; the adjusted HR for death associated with thyroiditis changed from 0.47 (95% CI, 0.44–0.53) in a time-independent model to 0.80 (95% CI, 0.71–0.89) in a time-varying model; three- and six-month landmark analyses also attenuated the association (21). These findings illustrate substantial immortal time bias. Residual confounding, cancer-specific heterogeneity, and the absence of a non-ICI comparison preclude interpreting thyroiditis as proof of treatment benefit or extrapolating the pooled estimate to gastrointestinal cancers. Future studies should distinguish thyroid phenotypes, model onset time, and report landmark exclusions and surveillance frequency.

### 2.3 Sex hormones

Sex steroids, including estrogens, progesterone, and androgens, have cell- and context-dependent effects on immune signaling and tumor biology. Nevertheless, biological sex, gender-related exposures, circulating hormone levels, and estrogen/androgen receptor expression are distinct variables, and the differences in cancer incidence or treatment outcomes between men and women cannot be fully explained on the basis of hormones.

Receptor-dependent effects may differ between malignant cells and immune populations, and according to ligand concentration and activation state. Estrogen receptor positivity in a tumor is not a surrogate measurement of circulating estradiol, nor does it establish the predictive value of serum estrogen. Direct clinical evidence linking circulating estrogen to differential ICI benefit in CRC remains insufficient. Guan et al. showed that androgen receptor activity within CD8^+^ T cells can suppress interferon (IFN)-γ expression and limit checkpoint blockade efficacy in experimental models of prostate cancer (32). This mechanism justifies investigation of cell-specific androgen signaling, but does not indicate that men with gastric cancer respond less favorably due to higher levels of circulating androgens. No validated serum estrogen or androgen cutoff for gastrointestinal ICI selection has been established so far. A 2026 pan-cancer single-cell analysis integrating 468 samples from eight tumor types likewise reported heterogeneous, tissue-dependent associations between sex-linked transcriptional programs and immune-cell states (33). Hormone-response signatures in such datasets are expression-based proxies, not measurements of circulating hormone concentrations. These findings support recording sex-related biological variables but do not validate serum estrogen or testosterone as gastrointestinal ICI selection markers.

Studies of sex-related differences should measure the proposed exposure directly and consider age, menopausal status, exogenous hormones, adiposity, disease burden, and treatment regimen. Hormonal concentrations and tumor or immune-cell receptor expression should be analyzed separately before testing their interaction. Endocrine manipulation to enhance ICI efficacy in gastrointestinal cancer remains investigational.

## 3 Role of nutritional biomarkers in ICI therapy for gastrointestinal tumors

Nutritional status plays a crucial role in the treatment tolerance and prognosis of cancer patients. Gastrointestinal obstruction, malabsorption, reduced intake, systemic inflammation, and treatment toxicity may coexist. Therefore, nutritional assessment requires clinical information on intake, weight loss, muscle mass, and function, rather than reliance on a single circulating protein (34, 35). In the following sections, we have distinguished indicators of clinical risk from candidate immune mechanisms and evidence that an intervention changes ICI outcomes. This approach is consistent with the 2025 GLIM update, which retains a diagnostic framework combining at least one phenotypic criterion – weight loss, low body mass index, or reduced muscle mass – with at least one etiologic criterion, namely reduced intake/assimilation or inflammation/disease burden (36). Serum albumin is not a substitute for this multidimensional assessment.

### 3.1 Albumin

Albumin, the most abundant protein in the blood, is mainly produced by the liver. While it maintains osmotic pressure and molecular transport, serum level of albumin is not a direct measure of protein intake or body protein stores. The ASPEN position paper emphasizes that albumin and prealbumin primarily characterize inflammation and adverse-outcome risk, rather than diagnose protein-energy malnutrition (35). Inflammation-related changes in synthesis and capillary permeability, hepatic dysfunction, renal or gastrointestinal protein loss, and fluid balance can all contribute to hypoalbuminemia (14). These processes should be assessed alongside nutritional history. It remains to be determined whether albumin is associated with treatment outcomes, which biological processes explain that association, and whether an intervention can improve outcomes. Low albumin may accompany inflammatory catabolism or advanced disease, without establishing impaired immune-cell protein synthesis as the causal pathway. **Figure 3** shows the determinants and interpretation of albumin levels. Nutritional support may address inadequate intake, whereas albumin infusion or an increase in its measured concentration does not itself demonstrate restoration of anti-tumor immunity (14, 35). Furthermore, low albumin may be indicative of increased catabolism of therapeutic IgG antibodies rather than direct immune failure (37). In a 2026 pharmacokinetics study, longitudinal albumin measurements were used to reproduce cachexia-related changes during durvalumab clearance (38). This provides a mechanistic modeling framework, not proof that albumin infusion improves drug exposure or clinical benefit. Treatment response can itself alter catabolism and clearance, thus complicating causal interpretation of albumin changes during treatment. The prognostic relevance of albumin-based measures also extends beyond ICI-treated populations. In 455 patients undergoing curative gastric cancer resection, Liu et al. associated a higher preoperative CRP/albumin ratio with poorer OS (39). This surgical cohort supports a general inflammatory prognostic interpretation, without establishing ICI-specific predictive value.

**Figure 3:**
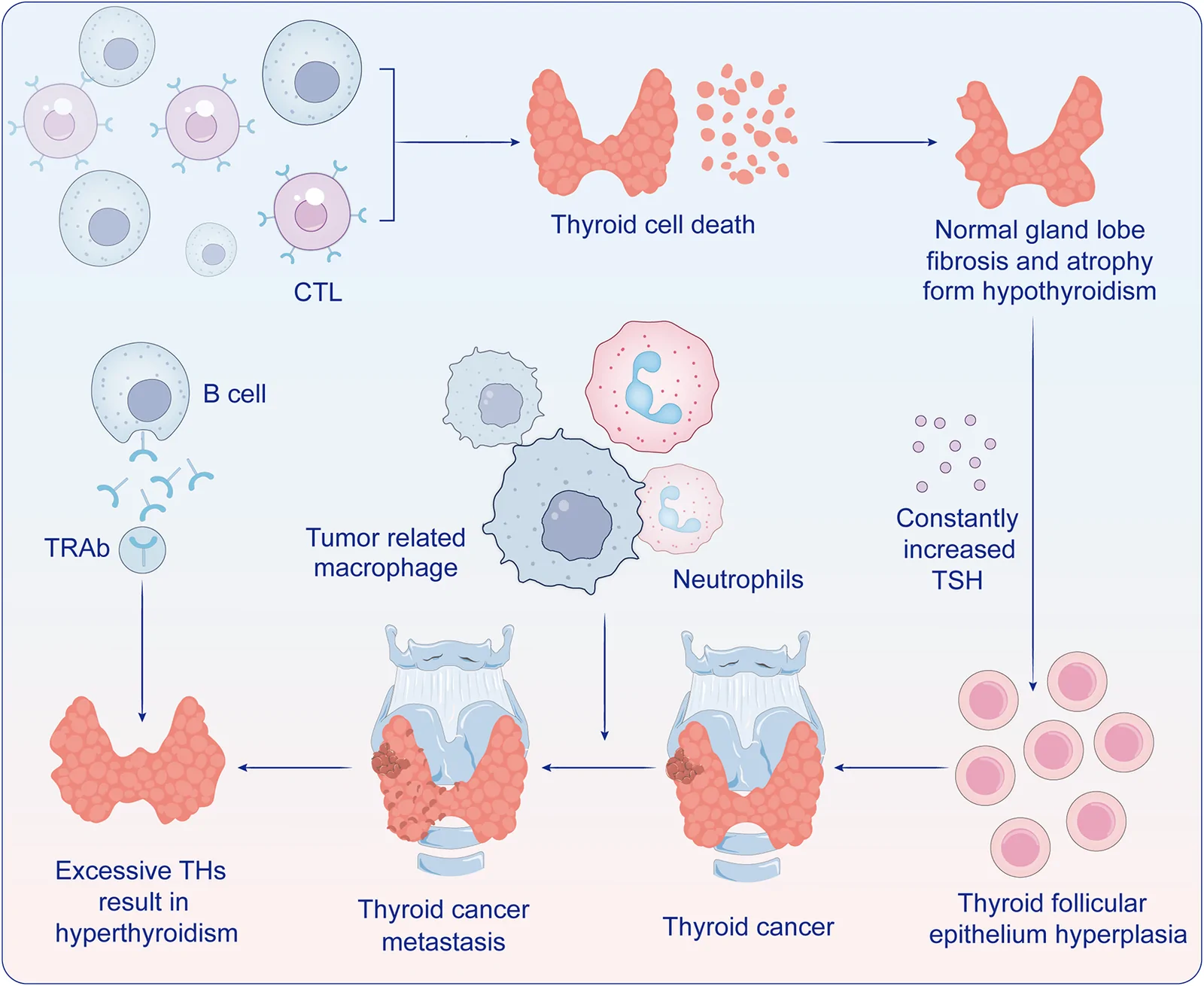
Immune checkpoint-mediated immunosuppression in the microenvironment of gastrointestinal tumors. This original schematic depicts an immunosuppressive tumor microenvironment in which regulatory T cells and CTLA-4-associated inhibitory signals restrict T-cell activation. The PD-1/PD-L1 axis further suppresses effector T-cell activity and facilitates tumor immune evasion. These pathways provide the biological rationale for immune checkpoint blockade in gastrointestinal cancers, although therapeutic responsiveness is influenced by tumor molecular subtype and microenvironmental context.

The gastrointestinal evidence is predominantly observational. Sato et al. retrospectively evaluated 278 previously treated patients with unresectable or recurrent gastric cancer receiving nivolumab. Pre-treatment albumin >3.5 g/dL was associated with longer OS after multivariable adjustment (HR, 0.688; 95% CI, 0.478–0.991) (16). However, this does not establish a universal 35 g/L threshold or a treatment–albumin interaction. Lee et al. studied 35 gastric cancer patients with peritoneal metastases: a baseline prognostic nutritional index (PNI) ≥40 was associated with longer PFS and OS, but the cohort was small and had no non-ICI control (40). PNI combines albumin and lymphocyte count, and must not be interpreted as albumin alone. Study-level estimates and limitations are summarized in **Table 1**.

Wang et al. subsequently examined 195 patients with HER2-negative advanced gastric adenocarcinoma, including 178 with pMMR and 17 with dMMR disease (41), and most had received anti-PD-1 therapy with chemotherapy. Baseline PNI, measured within three days before treatment, was associated with PFS after adjusting for MMR and PD-L1 status (HR per unit increase, 0.949; 95% CI, 0.924–0.975). The median PNI used for group comparisons was 45.5, whereas the response-derived ROC threshold was 47.18; these thresholds serve different analyses and should not be treated as interchangeable clinical cutoffs. The absence of a non-ICI comparator and eligibility requiring at least three treatment cycles limit treatment-specific and baseline-risk inferences. Takegawa et al. compared 37 nivolumab-treated patients with 35 historical taxane-treated patients in an advanced esophageal cancer cohort. The patients predominantly had squamous-cell carcinoma, and PNI was assessed before treatment. Within the nivolumab cohort, low PNI was associated with worse OS, but the analyses were unadjusted and no formal treatment–PNI interaction was reported (17). A significant association in one cohort and a non-significant association in another are not evidence of different treatment outcomes. Neither these observations nor changes in albumin levels during therapy demonstrate that nutritional correction increases ICI efficacy. Toxicity analyses should report irAE incidence and severity as separate outcomes. In a larger retrospective ESCC cohort, Wang et al. evaluated 677 patients receiving anti-PD-1 therapy alone or with chemotherapy, radiotherapy, or both (42). A higher pre-treatment geriatric nutritional risk index (GNRI; study-derived cutoff, 91.5) was associated with longer OS (adjusted HR, 0.648; 95% CI, 0.532–0.789). However, treatment modality and performance status differed with GNRI, and there was no non-ICI comparator or formal treatment–GNRI interaction analysis. GNRI therefore remains a prognostic composite of albumin and body-weight information, not evidence that a nutritional intervention increases ICI benefit.

### 3.2 Vitamin D

Vitamin D is a fat-soluble prohormone involved in calcium homeostasis and immune regulation. Circulating 25-hydroxyvitamin D [25(OH)D] is used to assess vitamin D status and is distinct from the active metabolite 1,25-dihydroxyvitamin D3 (calcitriol). Experimental calcitriol exposure, circulating 25(OH)D, and oral cholecalciferol supplementation are not interchangeable interventions or measurements.

Vitamin D activity is context dependent. Piemonti et al. cultured human monocyte-derived dendritic cells (DCs) with granulocyte–macrophage colony-stimulating factor and interleukin (IL)-4, and found that calcitriol inhibited differentiation and maturation into effective antigen-presenting cells (43). Furthermore, Jeffery et al. showed that calcitriol and IL-2 increased FoxP3/CTLA-4 expression in activated human CD4^+^CD25^-^ T cells, and promoted their immunosuppressive activity while reducing production of inflammatory cytokines (44). Thus, vitamin D cannot be described generally as promoting DC maturation and Th1 differentiation while suppressing regulatory T cells. Conversely, Giampazolias et al. demonstrated microbiome-dependent enhancement of tumor immunity and checkpoint responses in mice with increased vitamin D availability (45). These findings involve different metabolites, target cells, experimental conditions, and disease settings; none alone establishes efficacy of supplementation during gastrointestinal ICI treatment. Recent experiments using human cell lines also suggest ambiguity. Romano et al. cultured healthy donor-derived peripheral blood mononuclear cells with 1,25-dihydroxyvitamin D3 at 10 or 100 nM doses for five days, and observed reduced IFN-γ responses and concentration-dependent changes in CD8^+^ T-cell PD-1 and CTLA-4 expression (46). These *in vitro* exposures cannot be equated with serum 25-hydroxyvitamin D concentrations or the effects of oral supplementation in ICI-treated patients. Tumor genotype may further condition vitamin D signaling. In ARID2-deficient gastric cancer models, Liu et al. linked reduced VDR expression to Wnt/β-catenin activation and showed that calcitriol suppressed metastatic phenotypes (47). These findings support genotype-dependent biological effects, but do not establish a circulating vitamin D threshold or benefit from supplementation during ICI therapy.

In a clinical study involving 200 patients with advanced melanoma receiving first-line nivolumab or pembrolizumab, Galus et al. measured 25(OH)D levels before and approximately 2-4 weeks after treatment. Among 199 classified patients, those with normal levels at baseline or after supplementation had an ORR of 56.0%, compared to 36.2% in patients with persistently reduced levels. The adjusted PFS HR was 0.65 (95% CI, 0.49–0.94), whereas the difference in OS was not significant (48). However, non-randomized supplementation, calendar-period differences, and grouping partly by post-baseline measurements limit causal inference. Therefore, these findings do not establish that vitamin D supplementation improves ORR in gastric or colorectal cancer patients undergoing ICI treatment. The distinction between nutrient status and therapeutic supplementation was also demonstrated in the 2026 SOLARIS phase III trial (49). Ng et al. randomized 455 patients with previously untreated metastatic CRC into high-dose or standard-dose vitamin D3 with mFOLFOX6 or FOLFIRI plus bevacizumab groups. High-dose treatment involved 8,000 IU/day for 14 days followed by 4000 IU/day, versus 400 IU/day in the control group. Median PFS was 11.8 months (95% CI, 10.3–13.3) versus 10.3 months (95% CI, 9.4–12.2; one-sided P = 0.25), without improvement in the primary endpoint. Nevertheless, this trial could not establish either benefit or lack thereof from vitamin D supplementation during ICI therapy.

Several studies have evaluated the possible impact of vitamin D supplementation and toxicity. Grover et al. reported an association between pre-treatment vitamin D supplementation and lower odds of ICI-induced colitis in retrospective melanoma cohorts of 213 and 169 patients, with adjusted odds ratios of 0.35 (95% CI, 0.1–0.9) and 0.46 (95% CI, 0.2–0.9) respectively (50). These findings were based on exposure history, not a validated serum threshold or randomized prophylaxis trial. Treatment of documented deficiency may be indicated for established clinical reasons, but neither gastrointestinal anti-tumor benefit nor irAE prevention can be confirmed on this basis. Vemulapalli et al. reported a different toxicity-associated observation in 179 patients who had already developed immune-mediated colitis (51). Serum vitamin D ≤40 versus >40 ng/mL was associated with more frequent grade ≥2 colitis (48.0% versus 28.6%; P = 0.034). Measurements were taken within six months before or after ICI initiation, and the adjusted estimates for this severity comparison were not reported. It is difficult to draw inferences regarding incident colitis risk, supplementation efficacy, or anti-tumor response due to inclusion of only colitis cases and the broad sampling window. The study threshold should not be adopted as a universal deficiency definition.

### 3.3 Trace elements

Trace elements support enzyme activity, redox regulation, and immune-cell metabolism. However, circulating levels of these elements are influenced by inflammation, protein binding, organ function, and treatment-related losses. Evidence from cell cultures or engineered metal-containing nanomedicines should not be equated with evidence for a serum biomarker or oral dietary supplementation.

Iron is required for cellular metabolism, but its relation to anti-tumor immunity is not uniformly beneficial. Wang et al. showed that ICI-activated CD8^+^ T cells can promote iron-dependent lipid peroxidation and ferroptosis in tumor cells (52). In contrast, Ma et al. demonstrated that CD36-mediated lipid uptake and ferroptosis can impair tumor-infiltrating CD8^+^ T-cell function (53). These cell-specific effects argue against interpreting serum iron or ferritin as a direct measure of anti-tumor immune activity. Ferritin is also affected by inflammation, thereby limiting its interpretation without iron indices and clinical context. Evaluation and treatment of iron deficiency should therefore follow the hematological and nutritional needs of the patients.

Wang et al. conducted an exploratory single-center study of 98 patients receiving ICI-based combination treatment, including 47 with gastrointestinal cancers and 51 with lung cancer. Temporal training and validation cohorts included 68 and 30 patients respectively. Baseline levels of trace elements did not distinguish clinical benefit, whereas zinc level measured before the second ICI administration was associated with subsequent outcomes. The reported threshold was 14.2, but units differed between the abstract (μg/L) and results (μg/mL); it should not be transferred into practice without further validation (54). The findings are exploratory given the mixed tumor population, treatment-line imbalance, absence of gastrointestinal subtype-specific estimates, and post-baseline timing, and do not establish that zinc supplementation improves ICI efficacy. Experimental evidence also argues against high zinc intake being invariably beneficial. Narayan et al. found that high dietary zinc favored accumulation of Foxp3^+^ regulatory T cells and tumor growth in mouse models, and this effect was mediated by FOXO1 (55). Preclinical evidence suggests that zinc chelation can enhance anti-PD-1 activity, but does not justify zinc restriction or chelation in cancer patients.

Selenium contributes to selenoprotein-dependent redox control. Yao et al. showed that the selenium–glutathione peroxidase 4 (GPX4) axis protects follicular helper T cells from ferroptosis and supports antibody responses in experimental immunization and influenza-vaccination settings (56). A clinical link between circulating selenium level, PD-L1 expression, and ICI benefit in gastrointestinal cancer has not been established. Liu et al. identified an extracellular GPX4–ZP3 pathway through which GPX4 released from ferroptotic tumor cells impaired DC glycolysis and maturation, and T-cell priming in experimental systems (57). The antioxidant effects in lymphocytes and the extracellular suppression of antigen-presenting cells are therefore not interchangeable mechanisms. Neither observation establishes a serum selenium threshold or a selenium supplementation strategy for gastrointestinal immunotherapy.

Future clinical studies should specify assay calibration, units, specimen timing, reference intervals, supplementation, inflammatory status, and renal or hepatic dysfunction related to zinc, selenium, and iron. Correcting an established deficiency and selecting an anticancer treatment are different clinical objectives. Efficacy-directed supplementation requires randomized evaluation with relevant safety monitoring.

## 4 Interaction between hormone and nutritional biomarkers and their impact on ICI therapy

Hormonal changes, reduced food intake, inflammation, and altered metabolism often coexist in gastrointestinal cancer. Their correlations may reflect shared causes, reciprocal physiological effects, or consequences of disease progression. Distinguishing these possibilities is essential before interpreting a circulating biomarker as a mediator of ICI response.

### 4.1 Interaction between malnutrition and endocrine disorders

Cancer-related weight loss and muscle depletion are the result of reduced dietary intake and abnormal metabolism. Cachexia is not synonymous with simple starvation and may not be fully reversed by nutritional support alone (15). Severe illness can alter thyroid hormone conversion and binding proteins, while endocrine disease can adversely affect energy expenditure and physical function. Total hormone concentrations may therefore change without an equivalent alteration in biologically active free hormone. **Figure 4** summarizes these relationships without attributing sarcopenia or frailty to a single hormonal pathway.

**Figure 4:**
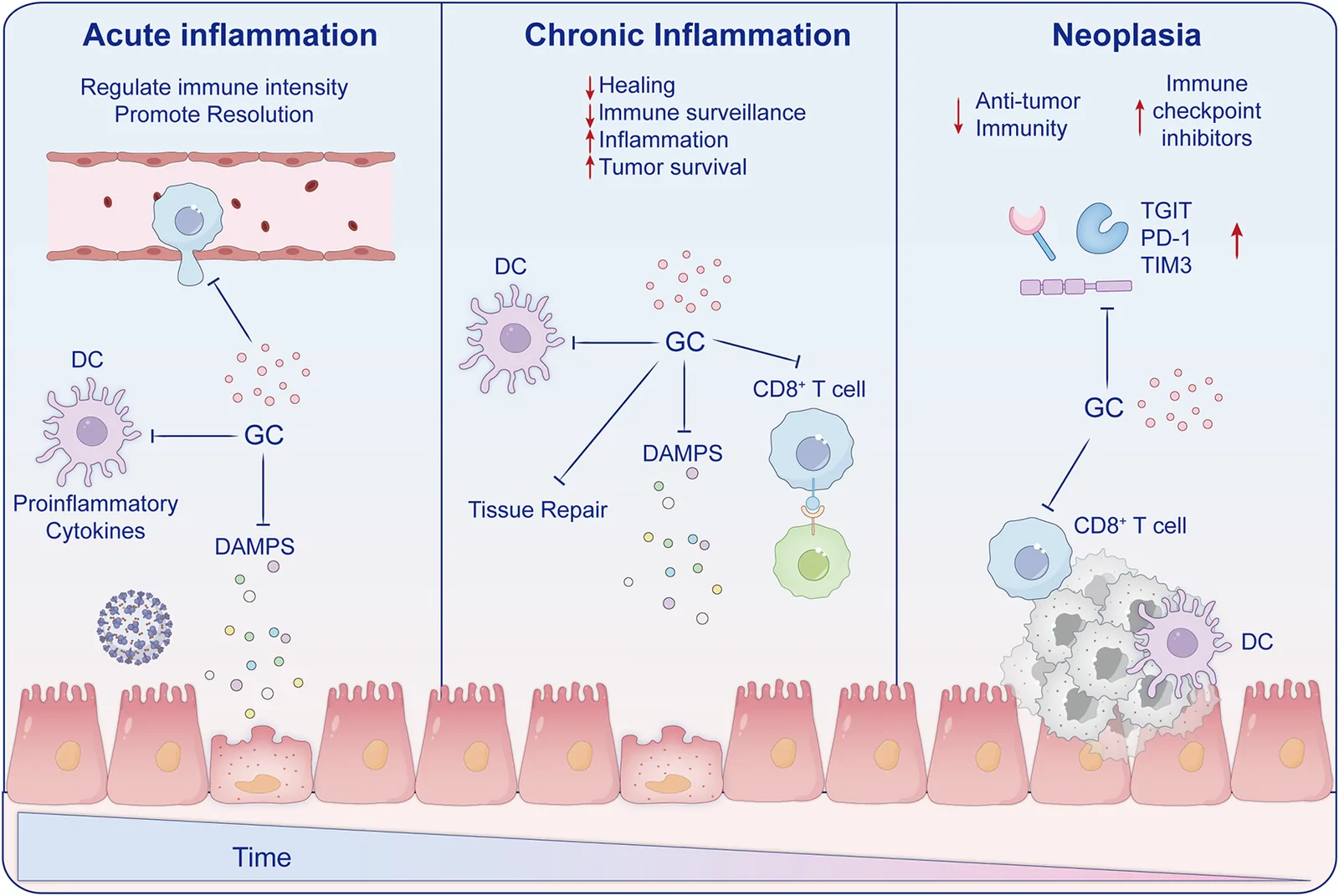
Interactions between frailty, malnutrition, and endocrine–metabolic dysregulation relevant to ICI treatment in gastrointestinal cancers. This original schematic illustrates how aging, lifestyle factors, comorbidities, and chronic inflammation may contribute to frailty, sarcopenia, malnutrition, and a catabolic state. These interrelated processes can impair functional reserve and increase vulnerability to adverse clinical outcomes during cancer treatment. Their potential associations with ICI tolerance and outcomes in gastrointestinal cancers remain hypothesis generating and require prospective validation.

Body-composition studies provide complementary, although still observational, evidence. Deng et al. retrospectively studied the pre-treatment L3 computed tomography measurements of 115 ICI-treated gastric cancer patients (58), and found that both sarcopenia (adjusted HR, 3.569; 95% CI, 1.808–7.045) and myosteatosis (adjusted HR, 3.172; 95% CI, 1.471–6.839) were associated with worse OS. Nevertheless, heterogeneous ICI regimens, incomplete molecular characterization, and data-derived muscle thresholds limit transferability, and these associations do not show that increasing muscle mass can improve ICI efficacy.

The timing of cachexia ascertainment also requires attention. In a 98-patient digestive-cancer cohort, Tao et al. classified cachexia using changes in body weight or psoas muscle area between treatment initiation and assessment after at least two cycles (59). Such an exposure incorporates on-treatment information and cannot be interpreted as a wholly pretreatment biomarker. Landmark or time-dependent analyses are needed to reduce bias arising from the requirement to survive and remain evaluable until reassessment.

Endocrine dysfunction can also complicate nutritional care: thyrotoxicosis increases metabolic demand, and GC excess can promote muscle catabolism and metabolic disturbances. These clinical mechanisms should be assessed independently of any hypothesized ICI-specific effect (30, 60). Nutritional intake, longitudinal weight change, muscle function, inflammatory markers, and endocrine diagnoses provide complementary information. Neither a cross-sectional association nor simultaneous normalization during treatment proves that correcting one biomarker can improve tumor control.

### 4.2 Systemic metabolism and tumor immunometabolic reprogramming

Systemic metabolic syndrome and intra-tumoral metabolic reprogramming are distinct mechanisms. Body mass index, glucose, lipids, and albumin provide different information, and cannot be treated as equivalent measures of immune competence. Rong et al. described alterations in glucose, lipid, and amino-acid metabolism in gastric cancer patients, which may support tumor growth and modify the local microenvironment (61). This framework connects nutritional physiology with tumor immunity, but circulating nutrient concentrations do not directly quantify metabolic flux in malignant or immune cells.

Glucose competition can restrict the glycolytic capacity of effector T cells and reduce IFN-γ production; Chang et al. demonstrated this mechanism and its modulation by checkpoint blockade in a mouse sarcoma model (62). Lipid metabolism may be more relevant in gastric cancer, as Zhang et al. reported that MFSD2A reduced the COX2–prostaglandin–TGF-β1 pathway, increased CD8^+^ T-cell activity, and enhanced anti-PD-1 responses in gastric cancer models, alongside associative human tissue findings (63). Amino acid remodeling, including glutamine utilization and serine/glycine-associated biosynthesis, supports tumor proliferation and redox balance, as reviewed by Rong et al. (61). The contribution of individual pathways to clinical ICI resistance remains context-dependent. These mechanisms support paired systemic and tumor-level studies rather than an assumption that supplementation, a higher albumin level, or absence of metabolic syndrome necessarily improves ICI efficacy.

Recent gastric cancer studies provide more specific links between metabolism and immune escape. Hu et al. showed that CLDN9-driven glycolysis increased lactate production and PD-L1 lactylation/stability, and suppressed CD8^+^ T-cell activity in experimental models (64). Chen et al. identified a related but distinct mechanism in *Helicobacter pylori*-associated disease: lactate-dependent H3K18 histone lactylation activated the HAS2–c-MYC–PD-L1 axis, and metabolic inhibition enhanced PD-1 blockade in a mouse model (65). These findings distinguish the signaling and epigenetic effects of a metabolite from nutrient availability alone. Metabolic signaling can also regulate checkpoint protein turnover. In CRC models, a study showed that penfluridol activated AMP-activated protein kinase (AMPK), promoted proteasomal PD-L1 degradation, and increased tumor suppression with anti-PD-1 in MC38-bearing mice (66). This pharmacological evidence supports a link between metabolic signaling and checkpoint activity, but does not validate circulating nutrient thresholds or establish clinical efficacy. A related mechanism involves checkpoint transcription. Wu et al. linked circRNA_0005075-dependent lactate production to H3K9 lactylation at the PD-L1 promoter and reduced T-cell-mediated killing in gastric cancer cocultures (67). However, the reported *in vivo* experiments used nude-mouse xenografts, limiting interpretation of adaptive immune effects; confirmation in immunocompetent models is necessary before inferring sensitization to PD-1 blockade.

Amino acid and lipid pathways show similar cellular specificity. Sun et al. found that loss of METTL7A stabilized the glutamine transporter SLC1A5, increased glutamine uptake by tumor cells, and restricted glutamine availability to CD8^+^ T cells in gastric cancer models (68). Jiang et al. described opposing macrophage and fibroblast contributions to nicotinamide metabolism that influenced CD8^+^ T-cell function and experimental anti-PD-1 responsiveness (69). Ning et al. linked cholesterol synthesis to a less inflamed MSS gastric cancer phenotype and demonstrated anti-PD-1 sensitization by simvastatin in tumor-bearing mice (70). These preclinical interventions concern defined intra-tumoral pathways; they neither validate serum albumin as a measure of tumor metabolic flux nor support routine amino acid supplementation, nicotinamide treatment, or statin initiation to enhance ICI efficacy.

### 4.3 Microbiota-derived metabolites within the nutritional endocrine and immune network

The intestinal microbiota provides a potential interface between dietary exposure, endocrine physiology, and immune regulation. Nutritional substrates and epithelial nutrient signaling influence microbial function; microbial metabolism in turn generates short-chain fatty acids (SCFAs), bile-acid derivatives, and tryptophan metabolites that act on host cells. Within this framework, a circulating nutrient or hormone may be associated with immunity through an intestinal pathway as well as through direct effects on immune cells (45, 71). Microbial composition alone cannot identify which pathway is active. **Figure 5** places microbiome-associated metabolism in the broader setting of treatment-related immune remodeling.

**Figure 5:**
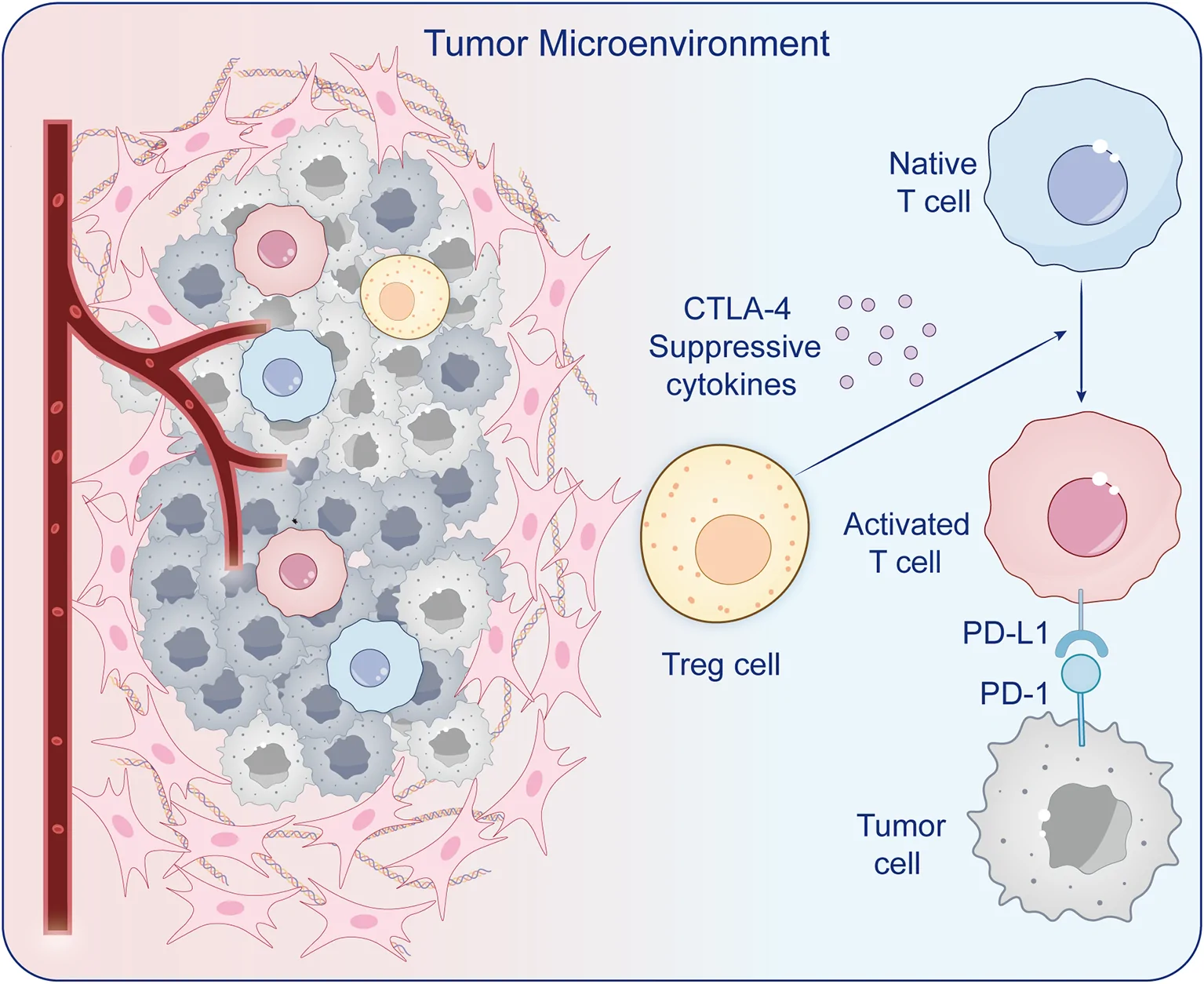
Chemotherapy-associated remodeling of the tumor immune microenvironment and its relevance to ICI-based therapy in gastrointestinal cancers. This original schematic illustrates how chemotherapy may induce immunogenic tumor-cell death and reshape the immune microenvironment in gastrointestinal cancers. It highlights changes in immune-cell composition, metabolic programming, and microbiome-associated metabolism that may enhance antitumor immunity. These processes may provide a biological rationale for chemoimmunotherapy, although their effects on ICI efficacy remain context dependent and require prospective validation.

Giampazolias et al. showed that vitamin D promoted *Bacteroides fragilis*-dependent anti-tumor immunity in mice by acting on intestinal epithelial cells (45). This illustrates an indirect pathway distinct from the effects of calcitriol on isolated immune cells. Spencer et al. found that diet and commercial probiotic exposure can differentially affect checkpoint response based on melanoma patient observations and experimental models (72). These findings do not support indiscriminate probiotic use or identify a universal favorable microbial profile for gastrointestinal cancer. Whether a defined microbial intervention improves gastrointestinal ICI outcomes requires direct clinical evaluation.

Microbial fermentation also links nutrient exposure to endocrine signaling. Tolhurst et al. showed that SCFAs stimulated glucagon-like peptide-1 secretion through free fatty acid receptor 2 (FFAR2) in experimental colonic systems (73). A separate mechanism involves sex hormones: transfer of male-associated microbiota increased testosterone and produced androgen-receptor-dependent protection from autoimmunity in nonobese diabetic mice (74). These observations establish experimentally testable connections between microbiota, nutrition, and hormones. Their contribution to ICI efficacy in gastrointestinal cancer has not been demonstrated, and an enteroendocrine or autoimmune phenotype cannot be assumed to predict tumor response.

Microbial modification of host bile acids provides another connection to immune differentiation. Hang et al. found that 3-oxolithocholic acid inhibited T-helper-17 differentiation through RORγt, whereas isoallolithocholic acid promoted regulatory T-cell differentiation through mitochondrial signaling and Foxp3 induction (75). Together with the vitamin D-dependent epithelial pathway described above (45), these findings show how nutrient-responsive intestinal physiology and microbial metabolites can converge on immune-cell states. Whether this convergence favors tumor control depends on the metabolite, responding cell population, and treatment context.

Several studies have implicated an immunomodulatory role of microbial tryptophan metabolites in gastrointestinal cancers. Jia et al. showed that indole-3-propionic acid influenced Tcf7-associated chromatin acetylation and progenitor-exhausted CD8^+^ T-cell states, and enhanced anti-PD-1 activity in experimental tumor models (71). Yang et al. subsequently demonstrated that oat-β-glucan altered *Faecalibacterium prausnitzii*-derived butyrate and indole-3-propionic acid, with enhanced anti-PD-1 activity in mice (76). The effects of microbes and their metabolites should not be interpreted as proof that dietary supplementation improves survival during ICI treatment. Metabolite effects are also checkpoint dependent. Coutzac et al. associated higher circulating butyrate and propionate with reduced benefit from CTLA-4 blockade in melanoma cohorts, and demonstrated suppression of dendritic-cell activation and antitumor T-cell responses by butyrate in mice (77). Consequently, SCFAs should not be classified uniformly as favorable, nor should fecal metabolite abundance be equated with systemic exposure or activity within the tumor.

Direct observations of gastric cancer patients suggest an influence of treatment regimen. Han et al. analyzed 279 stool specimens from 117 patients with HER2-negative advanced gastric/GEJ adenocarcinoma receiving chemotherapy, PD-(L)1 blockade, or their combination at baseline and during treatment; a separate 101-patient cohort was used for validation (78). Response-associated microbial signatures differed between the treatment groups. Since treatment allocation was not randomized and a formal treatment–microbiome interaction was not established, these findings cannot identify a universally favorable microbial taxon or justify substituting microbiome testing for established tumor biomarkers.

Interventional gastrointestinal evidence is emerging, although attribution remains difficult. RENMIN-215 enrolled 20 patients with refractory MSS metastatic CRC after at least two prior treatment lines. Fecal microbiota transplantation (FMT), tislelizumab, and fruquintinib produced an ORR of 20.0% (95% CI, 5.7–43.7) and median PFS of 9.6 months (95% CI, 4.1–15.1) in a single-arm phase II trial (79). The follow-up report screened 94 real-world patients and matched only 10 trial participants to 16 controls; an OS association favored the study regimen, whereas the PFS difference was not significant (80). Nonrandomized comparison and the antiangiogenic combination prevent isolation of the FMT contribution. These findings complement the gastric cancer feasibility data in Section 5.2, but do not establish microbial metabolites as validated mediators of clinical benefit.

Testing this integrated framework requires dietary and medication histories alongside paired stool, blood, and, when feasible, tumor measurements. Microbial pathway profiling and targeted metabolite quantification can assess whether changes in nutrient or hormone status precede changes in immune function. Stool collection, storage, intestinal transit, and sequencing or analytical batches require documentation; functional measurements should not be inferred solely from the relative abundance of a candidate taxon. The STORMS reporting framework supports transparent microbiome study reporting (81), while prospective perturbation and mediation studies are still needed to distinguish a causal pathway from shared effects of disease or treatment. Specimen choice also affects microbial profiles: Shen et al. detected differences between fecal and intestinal lavage samples from 30 CRC patients and 25 controls (82). Although this study did not assess ICI response, it highlights the need to validate microbiome signatures for each specimen type.

## 5 Clinical application of hormone and nutritional biomarkers in ICI therapy for gastrointestinal tumors

### 5.1 Construction of prognostic models and patient stratification

Host biomarkers may complement established clinical and tumor factors in prognostic research. However, a prognostic score requires a traceable source, a pre-specified endpoint, and appropriate validation. Combining correlated laboratory measurements does not inherently improve prediction. A model developed in one gastrointestinal cancer should not be extrapolated to another without accounting for molecular subtype, treatment setting, and baseline risk.

Tumor-derived signatures provide a complementary level of assessment. Gao et al. integrated single-cell and bulk transcriptomes to develop an immune-cell-associated prognostic model for esophageal adenocarcinoma (83). Survival discrimination alone does not establish ICI-specific treatment benefit; studies combining such signatures with circulating hormonal and nutritional markers must demonstrate incremental value over established clinicopathological predictors.

Clinical interpretation also requires analytical comparability. Routine endocrine and chemistry assays are available, but proposed ICI thresholds have not been harmonized across platforms or sampling conditions. In a Vitamin D Standardization Program comparison, only half of 32 ligand-binding assays met the prespecified mean-bias criterion of ±5% (84). Studies should therefore report assay platform, calibration and quality control, units, and sampling conditions, and retain the same method for longitudinal assessment. Morning cortisol must be interpreted with recent steroid exposure and binding-protein changes, while albumin requires consideration of fluid balance and inflammation. Microbiome signatures additionally depend on specimen handling, sequencing method, and a reproducible analytical pipeline (81). A locally optimized threshold cannot be transferred to another center without examining these sources of measurement variation.

**Table 2** summarizes verifiable examples of hormone and nutritional biomarkers. Albumin-based gastric scores and the zinc/CRP nomograms have limited validation. Li et al. developed the Endocrine–Nutritional Immunotherapy response score (ENI-Score) consisting of morning cortisol level, PNI, prealbumin, neutrophil-to-lymphocyte ratio, and CRP/albumin ratio in a multicenter cohort of 738 gastric cancer patients undergoing neoadjuvant immunochemotherapy and surgery (85). Although discrimination and calibration were reported, this score can only predict pathological response within a treated population, not the incremental benefit of adding an ICI. Moreover, model selection considered external performance, and inclusion required completion of surgery; these features limit the independence of validation and applicability to all patients starting treatment.

Model reporting should include the full equation, measurement window, missing-data strategy, event count, discrimination with uncertainty, calibration slope/intercept and plots, and performance in independent external populations. Apparent accuracy in the development sample is insufficient. Validation should compare a pre-specified clinical/tumor model with and without the host markers, address correlated inputs, and avoid using the test cohort for algorithm selection (86). Decision-curve analysis may describe potential utility, but a prospective impact study is needed to show that model-guided care improves outcomes. Dynamic models require pre-specified prediction times and appropriate handling of progression, death, and missing follow-up samples.

Single markers also have limited biological specificity: low albumin, altered cortisol, or a reduced trace-element concentration can arise from several conditions unrelated to ICI resistance. For example, the zinc classifier summarized in **Table 1** had a training specificity of 41.86%, despite a sensitivity of 100%; validation specificity was only 37% in a small mixed-cancer cohort (54). Such performance is inadequate for excluding a patient from ICI treatment and has not been validated separately in gastrointestinal cancers. Candidate thresholds should be prespecified and evaluated for a defined outcome and time horizon, with sensitivity, specificity, and predictive values reported with uncertainty. Predictive values will change with outcome prevalence. For survival models, time-specific calibration and net clinical benefit are also required; a high AUC alone does not establish a useful treatment rule (86, 87).

The original TRIPOD framework (88) has been superseded by TRIPOD+AI, which applies to both regression and machine-learning prediction models (86). Reporting completeness should be distinguished from methodological quality: PROBAST+AI separately addresses quality, risk of bias, and applicability across data sources, predictors, outcomes, and analysis (89). These frameworks are relevant to endocrine–nutritional signatures regardless of algorithm complexity.

Internal resampling estimates optimism within the development setting; a random split of the same cohort is not independent external validation (90). External evaluation should apply the fixed model to a relevant new population and examine both discrimination and calibration before any updating (87). This is particularly important when moving a host-marker model between MSI-H and MSS disease, across histological subtypes, or from later-line monotherapy to perioperative combination treatment.

Prediction and causal intervention require different evidence. A treatment–biomarker interaction can establish whether relative ICI benefit differs by marker status, but does not show that changing the marker changes that benefit (8). The latter question requires a controlled intervention with an appropriate clinical endpoint. Conversely, tumor response may improve intake, reduce inflammatory catabolism, and normalize albumin or hormone concentrations (14, 38), creating reverse causation in longitudinal analyses. Composite scores can improve risk estimation without identifying an actionable biological mechanism. Their proposed use should specify the clinical decision, the consequences of false-positive and false-negative results, and how a prospective impact study would test that decision (86).

### 5.2 Supportive interventions and their evidentiary limits

Clinical care should address endocrine disease and nutritional impairment when indicated, while keeping supportive benefits separate from unproven enhancement of ICI anti-tumor activity. The presence of an adverse prognostic marker does not indicate that altering the marker can affect prognosis, nor does it establish that the patient would fare better without an ICI.

Nutritional support may include dietary counseling, oral supplements, enteral nutrition, or parenteral nutrition according to intake, gastrointestinal function, and clinical need (34). Albumin alone should not determine the diagnosis of malnutrition or the choice of intervention. Vitamin and trace-element replacement should address documented deficiency or another established indication, with appropriate dosing and monitoring. The observational studies summarized above do not demonstrate that raising albumin, vitamin D, zinc, or selenium improves ICI-specific response or survival. Trials evaluating these interventions should measure patient-centered outcomes and treatment tolerance as well as biochemical correction.

Endocrine intervention should follow the diagnosis rather than a presumed anti-cancer effect. Overt hypothyroidism can require levothyroxine, whereas the transient thyrotoxic phase of destructive thyroiditis may need symptomatic management and monitoring rather than thyroid hormone replacement. Suspected central hypothyroidism warrants evaluation for adrenal insufficiency before thyroid hormone therapy is initiated. Controlled endocrine irAEs do not usually require permanent ICI discontinuation, although acute severe illness requires individualized management (30). Necessary physiological GC replacement must be distinguished from immunosuppressive dosing. Suppressing cortisol or manipulating sex hormones solely to improve gastrointestinal ICI efficacy remains investigational.

Dietary and microbiota interventions remain investigational as ICI efficacy modifiers, although direct gastrointestinal feasibility data are emerging. Zhang et al. evaluated healthy-donor fecal microbiota transplantation (FMT) with nivolumab in a single-arm phase I study of 10 patients with ICI-refractory gastrointestinal cancer, including eight with gastric cancer and two with CRC (91). After an initial oral FMT course, maintenance FMT was administered with nivolumab 3 mg/kg every two weeks for six cycles. Two patients, both in the gastric subgroup, had an objective response. The small, uncontrolled cohort cannot establish that FMT reversed resistance or improved survival. An ongoing phase I–II trial is evaluating the outcomes of antibiotic-assisted FMT followed by ICI-containing therapy in esophageal and gastric cancer patients (92). Regimen-specific randomized evaluation, and careful donor and infection-safety assessment are required before clinical adoption.

### 5.3 A pragmatic workflow for clinical assessment and follow-up

The immediate clinical role of hormonal and nutritional assessment is to identify conditions requiring supportive care and to contextualize treatment tolerance. **Figure 6** summarizes candidate biomarkers, whereas **Table 3** presents a proposed workflow that integrates endocrine and nutritional guidance with the interpretation of host-marker abnormalities (30, 34, 36). The workflow is intended for supportive assessment; its predictive accuracy and clinical impact have not been prospectively tested. ICI decisions should integrate the cancer indication, molecular findings, disease extent, functional status, comorbidities, and patient preferences.

**Figure 6:**
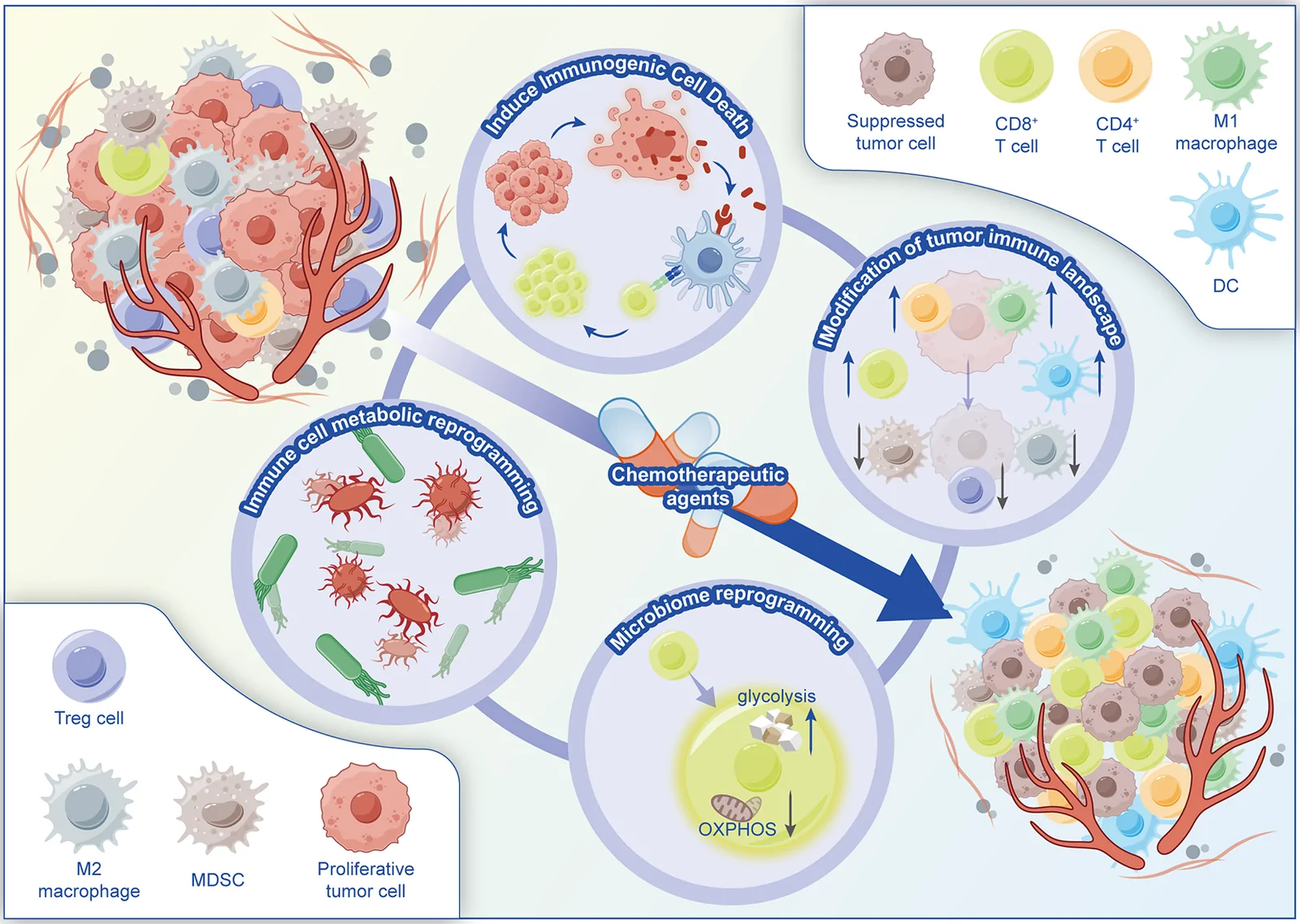
Candidate biomarkers associated with efficacy and atypical clinical outcomes of ICI therapy in gastrointestinal cancers. This original schematic categorizes candidate biomarkers according to their reported associations with treatment efficacy, irAEs, pseudoprogression (PsP), and hyperprogressive disease (HPD). The proposed markers include tumor- and host-derived features, such as MSI-H/dMMR status, PD-L1 expression, tumor mutational burden, circulating tumor DNA, inflammatory indices, and microbiome-related factors.

**Table 3:** Proposed workflow for endocrine and nutritional assessment during ICI therapy in gastrointestinal cancers.

Clinical step or finding	Assessment	Clinical action	Implication for ICI decisions
1. Before treatment	Establish stage, histology, indication-specific tumor markers, ECOG PS, prior therapy, and medication history.	Select the cancer-specific regimen. Record baseline thyroid tests, weight and intake, and interpret routine laboratory results in context (1–3, 6, 30, 34).	Hormonal or nutritional risk markers alone do not establish ICI ineligibility.
2. Nutritional risk or low albumin	Review weight loss, intake, muscle or functional measures, CRP, organ dysfunction, and protein loss.	Apply GLIM criteria; arrange dietetic assessment and indicated nutritional support. Reassess intake, weight, and tolerance (14, 34–36).	Correct identifiable nutritional problems. A rise in albumin does not demonstrate ICI sensitization.
3. Abnormal thyroid tests	Interpret TSH with free T4. With suspected central dysfunction, assess the adrenal axis before levothyroxine.	Treat confirmed endocrine disease according to its phenotype; use closer laboratory follow-up for evolving thyroiditis (30).	Stable, treated endocrine dysfunction generally permits ICI continuation. Acute severe illness requires individualized interruption.
4. Possible adrenal crisis	Urgently evaluate hypotension, vomiting, marked weakness, or other compatible signs; obtain cortisol/ACTH when feasible.	Begin emergency glucocorticoid treatment and appropriate supportive care without awaiting test confirmation (30).	Prioritize stabilization; the acute safety issue determines temporary treatment management.
5. During treatment	Reassess TSH/free T4 generally every 4–6 weeks; review symptoms, intake, weight, medications, and organ function.	Investigate new abnormalities and treat the identified cause. Assess tumor response through the established oncological pathway (30, 34).	Biochemical deterioration or normalization alone cannot diagnose resistance or confirm response.
6. Investigational risk score	Check the fixed model, assay requirements, prediction time, endpoint, calibration, and external applicability.	Use a high-risk result to inform supportive assessment or study stratification; evaluate decision consequences prospectively (86, 87).	Treatment exclusion and altered surveillance require validated clinical utility for the specific decision.

The clinical actions draw on endocrine and nutritional guidance (30, 34, 36); their integration with host-marker interpretation is proposed by the authors and has not undergone prospective validation. Severe irAEs require management according to established guidance (27, 30). ACTH, adrenocorticotropic hormone; CRP, C-reactive protein; ECOG PS, Eastern Cooperative Oncology Group performance status; free T4, free thyroxine; GLIM, Global Leadership Initiative on Malnutrition; ICI, immune checkpoint inhibitor; TSH, thyroid-stimulating hormone.

Before treatment, tumor MSI/MMR status and other indication-specific biomarkers should be assessed according to the relevant treatment pathway. Baseline thyroid tests and clinically indicated endocrine assessment provide a reference for toxicity monitoring (30). Nutritional assessment should include intake, weight trajectory, and functional or muscle measures (34). An unfavorable cortisol, albumin, vitamin D, or trace-element profile should prompt evaluation of its cause; it is not an established reason to withhold an otherwise indicated ICI. Broad hormonal or micronutrient panels for treatment selection require prospective evaluation.

An abnormal baseline result should lead to a defined diagnostic branch. Hypoalbuminemia warrants assessment of intake and weight loss together with CRP, hepatic function, renal or gastrointestinal protein loss, and fluid status (14, 35). Malnutrition assessment should then apply clinical criteria such as GLIM and determine the need for dietetic referral and nutritional support (34, 36). Discordant thyroid results require paired TSH/free T4 interpretation; low free T4 with an inappropriately low or normal TSH warrants pituitary and adrenal evaluation before levothyroxine. Symptoms suggesting adrenal crisis require urgent assessment and glucocorticoid treatment without waiting for confirmatory laboratory results (30). These branches address identifiable clinical conditions rather than classify ICI sensitivity.

During treatment, TSH and free T4 should generally be reassessed every 4–6 weeks, with earlier evaluation for new symptoms or a known abnormality (30). Weight trajectory, intake, and function should be reviewed during clinical visits (34). New biochemical abnormalities require interpretation with medication changes, organ function, and radiological assessment. Persistent abnormalities may reflect inflammation, malabsorption, toxicity, or progression, and cannot independently diagnose drug resistance. Similarly, biochemical normalization is not a surrogate for tumor response. Thyroid changes may identify an irAE requiring treatment, but their association with survival does not justify inducing or leaving toxicity untreated. Changes in ICI therapy should follow the overall clinical and oncological assessment rather than an unvalidated host-marker rule.

After treatment or surgery, surveillance should follow the cancer type, stage, treatment setting, and established follow-up pathway. Stable hormonal or nutritional biomarkers have not been shown to exclude recurrence or justify extending follow-up intervals. Abnormal values should trigger an appropriate clinical assessment without being interpreted as proof of recurrence. Biomarker-guided changes in surveillance intensity remain hypotheses for prospective clinical-utility studies.

Translation into routine decision-making will require reproducible assays, interpretable thresholds, independent validation, and evidence that acting on a result benefits patients. Multidisciplinary interpretation can support appropriate endocrine and nutritional care now, while avoiding premature treatment selection based on observational risk associations. Where an investigational score identifies high risk, a defensible immediate action is closer supportive assessment or research stratification. Using that score to omit ICI therapy, escalate an unproven intervention, or alter surveillance requires evidence specific to that decision and population (86, 87).

## 6 Challenges and prospects

The central challenge is to determine whether host biomarkers add clinically useful information beyond tumor biology and disease severity. Mechanistic plausibility and inexpensive measurement are valuable, but neither guarantees independent prognostic value or prediction of treatment benefit.

Priority limitations include retrospective selection, small event counts, heterogeneous regimens, missing molecular subtype data, variable sampling times, and data-derived cutoffs. Baseline inflammation, liver disease, protein loss, corticosteroid indication, and performance status should be considered explicitly. Treatment-emergent analyses must address immortal time bias, informative discontinuation, and the fact that patients with early progression contribute fewer repeated samples. In CRC, MSI-H/dMMR and MSS/pMMR cohorts require separate reporting; gastric and esophageal analyses should likewise retain molecular and histological distinctions. The stage, treatment-history, and medication variables detailed in Section 1.3 should be incorporated into study design and prespecified sensitivity analyses, with baseline exposures kept separate from changes arising after treatment begins.

Prospective cohorts should pre-specify assays and sampling windows, document concurrent treatments and supplements, and distinguish baseline risk from on-treatment change. Comparative datasets should test treatment–biomarker interactions rather than infer predictive value from within-arm significance. Randomized supplementation or endocrine-intervention trials would be needed to establish whether modification of a biomarker changes ICI efficacy. Deficiency correction, toxicity control, tumor response, survival, and quality of life should be treated as distinct endpoints.

External validation sample sizes should be planned for sufficiently precise estimates of discrimination, calibration, and where relevant, net benefit, using the expected event frequency and prediction distribution rather than an arbitrary train–test split or a generic events-per-variable rule (93). Adequate overall sample size does not remove the need to assess performance within clinically important molecular and treatment subgroups.

Paired blood and tumor profiling may clarify how systemic measurements relate to intra-tumoral biology. Spatial metabolomics, transcriptomics, and cell-resolved immune profiling could test whether nutrient competition, lipid handling, and amino-acid metabolism mediate the observed host-marker associations (61, 63). These approaches should be linked to well-annotated clinical cohorts and pre-specified hypotheses rather than used to infer causation from cross-sectional correlations. Immune recruitment provides a complementary experimental target. Researchers showed that a CCL19-expressing oncolytic influenza virus increased immune-cell infiltration and suppressed CRC growth in syngeneic mice (94). This offers a preclinical example of immune remodeling, without demonstrating ICI synergy or validating a circulating hormonal or nutritional biomarker.

Spatially resolved metabolic profiling may help refine this approach. Lan et al. identified lactate-associated epithelial and myeloid states in CRC and spatial colocalization of metabolically active epithelial cells with fibroblasts using single-cell, spatial, and bulk transcriptomics (95). Such compartment-specific observations provide a basis for mechanistic investigation, while transcriptomic scores remain proxies for metabolic activity and require prospective validation in ICI-treated CRC.

More complex algorithms are not necessarily more useful. Correlated inflammatory and nutritional measurements can duplicate information, and apparent performance can be inflated by model selection or data leakage. Transparent reporting, independent recalibration, and prospective evaluation of decision consequences are needed before artificial intelligence-derived host signatures can guide care (86, 89).

## 7 Conclusion

Hormonal and nutritional measurements provide clinically relevant information on the outcomes of ICI treatment in gastrointestinal cancer, mainly assessment of endocrine toxicity, nutritional needs, and general clinical risk. Albumin-based indices are predominantly prognostic, while evidence concerning endogenous cortisol and integrated host scores is emerging. Baseline thyroid function must remain distinct from treatment-emergent thyroiditis, and sex or receptor expression cannot substitute for circulating hormone measurements.

Vitamin D and trace elements have biologically plausible, context-dependent effects, but supporting clinical studies are often indirect or exploratory. Tumor immunometabolic research offers a basis for further investigation rather than a rationale for unvalidated supplementation or endocrine manipulation. The microbiota offers a mechanistic link between nutrition, endocrine signaling, and immunity, but gastrointestinal intervention studies have not yet isolated its contribution to ICI benefit. Host-marker interpretation must also account for disease stage, prior treatment, and concomitant medications. These markers should not currently replace established tumor biomarkers, determine ICI exclusion, diagnose resistance, or lengthen surveillance intervals. Their clinical value will depend on reproducible measurement, disease-specific validation, comparative treatment evidence, and demonstration that biomarker-guided action improves patient outcomes.
